# Post-traumatic growth in psychosis: a systematic review and narrative synthesis

**DOI:** 10.1186/s12888-021-03614-3

**Published:** 2021-12-06

**Authors:** Fiona Ng, Nashwa Ibrahim, Donna Franklin, Gerald Jordan, Felix Lewandowski, Fan Fang, David Roe, Stefan Rennick-Egglestone, Christopher Newby, Laurie Hare-Duke, Joy Llewellyn-Beardsley, Caroline Yeo, Mike Slade

**Affiliations:** 1 School of Health Sciences, Institute of Mental Health, University of Nottingham, Nottingham, UK; 2 Psychiatric and Mental Health Nursing Department, Faculty of Nursing, Mansoura University, Mansoura, Egypt; 3 NEON Lived Experience Advisory Panel, Nottingham, UK; 4 School of Medicine, Yale University, Connecticut, USA; 5 School of Psychology, University of Nottingham, Nottingham, UK; 6 Department of Clinical Psychology, Kwai Chung Hospital, Hong Kong, China; 7 Department of Community Mental Health, University of Haifa, Haifa, Israel; 8 School of Medicine, Institute of Mental Health, University of Nottingham, Nottingham, UK

**Keywords:** Post-traumatic growth, Positive changes, Psychosis, Systematic review, Narrative synthesis

## Abstract

**Background and objective:**

People with psychosis report experiences of highly traumatic events. Positive change or post-traumatic growth (PTG) can occur as a result of traumatic experiences. Yet there is limited attention on PTG in psychosis, possibly due to the negative impact of psychotic symptoms on functioning and quality of life. The aim of this review was to identify significant correlates and mediators of PTG in psychosis, and to develop a conceptual framework synthesising facilitators of PTG in psychosis.

**Method:**

Ten electronic databases were searched in seven languages, and five journals and grey literature were searched in English. Quantitative studies were eligible if examining correlates, mediators, or the temporal relationship between PTG and one or more variables. Qualitative studies were eligible if describing PTG arising from experiences of psychosis. Findings from quantitative papers were grouped by analysis method, with significant correlates, mediators, and temporal relationships descriptively reported upon. Narrative synthesis was conducted on findings in qualitative papers.

**Results:**

Thirty-seven papers were included. Significant correlates and mediators of PTG were identified. Mediators of PTG in psychosis included meaning in life, coping self-efficacy, core beliefs, and self-reported recovery. No studies describing the temporal relationship between PTG and psychosis were identified. The narrative synthesis identified seven facilitators of PTG in psychosis: Personal identity and strength, Receiving support, Opportunities and possibilities, Strategies for coping, Perspective shift, Emotional experience, and Relationships, giving the acronym PROSPER.

**Conclusions:**

Individuals with psychosis can be supported to grow from traumatic experiences. Clinicians can support PTG through the provision of trauma-informed care that supports positively valued identity changes. For researchers, the findings provide an evidence-based theoretical framework for conceptualising PTG, which can be validated through longitudinal cohort studies and underpin the development of new clinical interventions.

**Supplementary Information:**

The online version contains supplementary material available at 10.1186/s12888-021-03614-3.

## Introduction

Psychosis can be a highly traumatic experience with negative consequences on functioning and quality of life [1], such as increased rates of loneliness [2], and reduced rates of engagement in employment [3]. Current psychosis research has focused on delineating the mechanisms that contribute to the development and maintenance of the disorder [4, 5]. Interventions and service models have been developed primarily to reduce these negative experiences. Yet, research shows that people with psychosis also report positive changes associated with their experiences [6, 7]. Such changes can include self-discovery, developing sense of self, a greater appreciation in life, improved wellbeing and relationships, and spiritual engagement [7].

The most well-established model describing positive change following trauma is that of post-traumatic growth (PTG), which is defined as positive psychological changes that can arise following from the highly emotional struggle with a traumatic or stressful experiences [8]. PTG has been described as occurring in at least five areas: (1) increased appreciation for life, (2) more meaningful relationships, (3) increased sense of personal strength, (4) identifying new priorities, and (5) a richer existential and spiritual life. PTG has been documented following physical illnesses like cancer [9], natural disasters [10] and terrorism [11]; and has been documented across cultures and context [12].

PTG has been reported by people with first-episode psychosis (FEP). Moderate increases in PTG have been reported [13], with positive changes across three domains 1) intrapersonal (e.g. greater clarity of self), interpersonal (e.g. improved relationships) and 3) spiritual [14, 15]. Experience of positive change was supported through engagement with mental health services. However, the state of knowledge on PTG among persons reporting multiple episodes of psychosis, as well as persons who are not engaged in clinical services, remains unclear. Given that many people who experience a first episode of psychosis often experience additional psychotic episodes, and a high proportion of people who experience a psychosis recover through non-clinical interventions (i.e., peer support, hearing voices groups, etc.), this substantial knowledge gap precludes an understanding of how to develop interventions to support PTG in these contexts. There is a need to synthesise knowledge on what facilitates/predicts PTG following psychosis across a broader range of contexts beyond the first episode.

To address this substantial knowledge gap, this systematic review aimed to examine predictors and perceived facilitators of PTG following psychosis, across the psychosis continuum, as described within quantitative, qualitative and mixed-methods studies. With the aim to establish causality using Bollen’s criteria, the objectives were to establish characteristics associated with PTG in psychosis (correlation; Objective 1), to identify associations with PTG that are independent of established factors that predict of PTG (isolation; Objective 2), to identify temporal relationships between PTG and psychosis (direction; Objective 3), and to characterise facilitators of PTG in psychosis from qualitative studies (Objective 4).

## Method

The systematic review protocol was conducted in accordance with the Preferred Reporting Items for Systematic Reviews and Meta-Analyses (PRISMA) guidelines [16] and prospectively registered on PROSPERO (CRD42020176403) in March 2020. This work will inform the process evaluation of three clinical trials in the NEON Study (ISRCTN11152837; ISRCTN63197153; ISRCTN76355273). The NEON Study is evaluating whether access to people’s real-life stories of mental health recovery is helpful for people affected by mental health problems (e.g. psychosis) and informal carers.

### Search strategy

Medical Subject Headings and key word searching was used to identify papers. Search terms included: “post-traumatic growth”, “positive change”, “benefit finding” or “stress related growth” combined with terms related to the population “psychotic disorder” and “schizophrenia”. The full search strategy for Medline is presented as Online Supplement 1, which was specialised to each of the other databases. The search strategy was developed by nine researchers in consultation with two information specialists. Information sources for studies published in English was searched between 1995 and April 2020. Whilst information sources for studies published in languages other than English was searched between 1995 and June 2020. The search was completed between April and June 2020.

### Information sources

Six information sources were searched: (1) electronic databases (n=10): Medline (English, French, Italian), Embase, PsycINFO (English, French, Italian), Scopus, Web of Science, Pilots, CINAHL, ZB Med Informationszentrum Lebenswissenschaften (German), Al Manhal (Arabic), and Chinese Academic Journals Full-Text Database (Chinese); (2) academic journals publishing papers written in Hebrew (n=2); *Sichot* and *Chevra Ve’Revacha*, (3) hand searching the table of contents of key journals (n=3) based on expert recommendations of *Psychiatric Services*, *Psychosis*, and *Journal of Mental Health;* (4) web-based searching (n=3) of *Google Scholar*, *ResearchGate* and *Academia.edu;* (5) forward citation tracking of all included studies using Scopus and backward citation tracking was manually completed on the reference lists of all included studies; (6) the list of included articles was sent to experts (n=3) to identify further eligible studies. Experts had expertise in psychosis and mental health services research. The searching of information sources 3 to 6 was only conducted in English. Given the language expertise of the research team, studies were included if published in Arabic, Chinese, English, French, German, Hebrew or Italian.

### Eligibility criteria

Empirical studies which examined PTG among people with experience of psychosis were eligible. To incorporate findings across the psychosis continuum [17], both diagnostic (e.g. having received a diagnosis of a psychotic disorder) and dimensional (e.g. spiritual emergence, hearing voices) experiences of psychosis were included. Therefore, both clinical populations and people with psychosis-like experiences were included in the review. Clinical diagnoses of psychosis eligible for review included; schizophrenia, schizoaffective disorder, schizophreniform disorder, delusional disorder, bipolar disorder or other mental health problems with psychotic features (e.g. depression with psychotic features). No restrictions on time since first experience of psychosis or whether participants have used mental health services previously were applied. To maximise robustness of findings, the search was not limited by specific populations, therefore studies describing PTG in people with psychosis in forensic settings or people over 65 years with psychosis were included in the review. This allowed for a broad spectrum of perspectives to be included in the review, which in turn improved the generalisability of findings. Eligible designs included quantitative, qualitative or mixed-methods designs. To avoid the inclusion of case studies, only studies with a sample size greater than three was included. A sample size of greater than three was chosen to allow for in-depth qualitative studies with a small sample to be included.

Different eligibility criteria for quantitative and qualitative papers were used to identify papers that are specific to the objectives of the study. Quantitative papers were eligible if the results described an association or temporal relationship between PTG and one or more variables, using a psychometrically validated measure of PTG. Qualitative findings from papers were included if they discussed specific facilitators of PTG arising from experiences of psychosis. Studies where aspects of PTG were reported as part of a wider focus, for example relating to recovery in psychosis, were also included. In this review, it is conceptualised that PTG is related to personal recovery, however PTG has a more narrow focus on the positive psychological changes that occur following trauma, particularly surrounding the reconstruction of a person’s sense of self and making meaning from experiences. Findings from mixed-methods studies were split into quantitative and qualitative designs and assessed using the corresponding criteria. Systematic reviews, books, book chapters, commentary pieces and conference abstracts were excluded from the review.

### Study selection and data abstraction

The search was completed by eight researchers (FN, NI, DF, GJ, FL, DR, MS, FF). For papers written in English, after the removal of duplicates, titles and abstracts of relevant papers were screened by FN, with 10% randomly selected independently screened by NI. Full-text screening was completed by FN and NI. Lack of consensus about inclusion was resolved by discussion with a third analyst (DF). Three analysts (FN, NI and DF) independently extracted data from the included studies, and double coded the same 10% of included papers (concordance=100%). For papers written in a language other than English, the search and screening process was completed by one bilingual analyst, with 10% of identified papers second screened by a second analyst with relevant language skills. Data were independently extracted by one analyst. Average concordance between analysts across all languages was 94%. Data were first extracted from papers published in English on 26th April 2020. Papers in languages other than English followed the same procedure and the search and screening was conducted by a researcher with expertise in the specific language. All papers were screened using Endnote X9.

Data extracted from all included studies comprised; study characteristics (publication language, country, aims, design, participants, sample size, setting, and inclusion criteria), participant characteristics (age, gender, ethnicity, marital status, diagnosis, working status), treatment and support experiences (type of treatment or support received, length of treatment), measures and data analysis, data on variables examining the relationship with PTG, and subjective experiences of PTG.

### Quality assessment

The Mixed Methods Appraisal Tool (MMAT) was used to critically appraise all included studies, with separate criteria applied for the different study methodologies [18]. MMAT does not allow for the exclusion of studies based on the assessment of their quality, however to conduct a sensitivity analysis, studies were assigned a score out of seven indicating the number of features present in the paper [19]. The presence of a feature on the MMAT was scored as one, whilst the absence or inability assess a feature resulted in a score of zero. Studies scoring below 50% were regarded as of low methodological quality.

### Analysis

To explore objectives 1 to 3, quantitative studies were analysed using Bollen’s causality criteria: association (objective 1), isolation (objective 2) and direction (objective 3) [20]. Studies investigating cross-sectional correlates were grouped to identify significant correlations between PTG and outcomes, whilst studies investigating PTG cross-sectional correlates whilst controlling for other variables were grouped to identify significant mediators of PTG. Longitudinal studies investigating temporal relationships between PTG and its covariates were grouped to examine direction. A significance level of 0.05 was used.

For objective 4, all qualitative data from included studies was analysed using a narrative synthesis approach to develop a conceptual framework of facilitators of PTG in psychosis. An adapted three-stage narrative synthesis approach [21] was used. The first stage of the Popay and colleagues’ [21] approach was omitted as decisions about scope and eligibility of studies had been established. The first stage of this review involved a preliminary thematic synthesis of the 9 section of each included paper to identify facilitators of PTG arising from psychosis. Participant responses which indicated positive change were counted as a theme. Findings were then tabulated and thematically analysed to identify those characteristics facilitating the experience of PTG. Similar characteristics were then clustered into themes using an iterative process consulting analysts from a range of academic backgrounds including clinical psychology, health services research, sociology, mad studies, and computer science. Vote counting was used as a descriptive tool to indicate the frequency of each theme. This has previously been used in other systematic reviews adopting a narrative synthesis [22]. For stage 2, the relationships within and between studies were explored. Specifically, the conceptual framework (stage 1) was compared between those studies recruiting participants through mental health services (e.g. community mental health) with other routes (e.g. Hearing Voices Movement or clubhouses), and between studies involving first-episode samples compared with samples with multiple or long-term episodes of psychosis. In stage 3, a sensitivity analysis of the conceptual framework (stage 1) was conducted with studies deemed to be high quality to determine the robustness of the synthesis.

The research team had expertise in a diverse range of academic and clinical perspectives including mental health services research, survivor research, critical qualitative health research, qualitative research in recovery, statistics, and clinical psychology. Some members of the research team also identify with having lived experience of mental distress or mental health problems. These varying perspectives allowed for a more balanced perspective in the interpretation of findings.

## Results

### Included studies

**Fig. 1:**
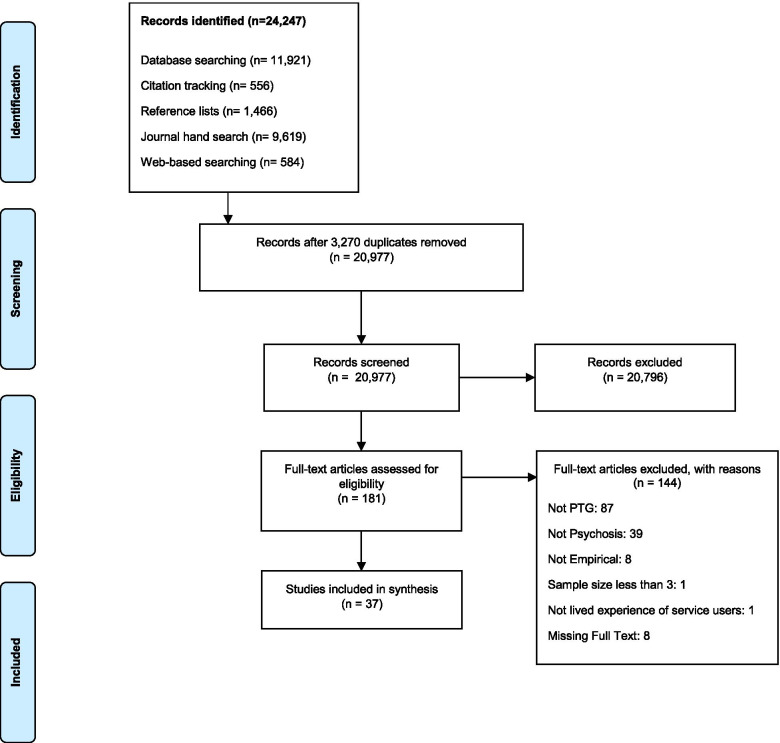
Flow diagram of the study selection process

The search identified 20,977 studies, with 37 papers meeting the inclusion criteria. The PRISMA flow diagram is shown in Fig. 1.

The data abstraction table including a full reference and paper identification number for each paper is shown in Online Supplement 2. The 37 papers comprised 34 distinct studies, involving a total of 709 participants (682 service users, 8 carers, 19 mental health workers). All papers were published in English (n=37), and consisted of qualitative (n=30), quantitative cross-sectional (n=4), and mixed-methods (n=3) study designs. No intervention studies were identified. Participants were recruited through clinical services (n=27), other routes (n=9) or both (n=1). Most (n=31) studies involved participants with multiple episodes of psychosis, with the remaining involving first-episode psychosis (n=6). Studies were conducted in 12 countries, including United Kingdom (n=10), Australia (n=7), Israel (n=4), and Hong Kong (n=3). All quantitative papers measured PTG using the post-traumatic growth Inventory (PTGI) [23]. The quantitative findings in one study with a mixed-methods design (paper #31) did not meet the inclusion criteria and therefore were not analysed. Using the MMAT, only two studies (papers #31 and #12) were considered of low methodological quality.

### Objective 1: characteristics associated with PTG

Objectives 1 and 2 were analysed using data from three samples reported across six papers, which here are called sample 1 (reported in papers #9, #10, #11) sample 2 (#14) and sample 3 (#7, #37). No covariates were evaluated in sufficient studies to allow meta-regression analysis. Significant cross-sectional correlates of PTG are shown in Table 1.

**Table 1:** Correlates of posttraumatic growth (n=6)

	Sample 1			Sample 2	Sample 3	
**Paper Number**^a^	**9**	**10**	**11**	**14**	**7**	**37**
**Sample Size**	121	121	121	34	94	94
**PTG Scale used**	PTGI	PTGI	PTGI	PTGI	PTGI	PTGI
**Objective 1: Association**						
Symptomatology (PANSS total)		- - -	- - -			
Positive Symptoms (PANSS sub-scale)		-	-			
Negative Symptoms (PANSS sub-scale)		- - -	- - -			
General Psychopathology (PANSS sub-scale)		- - -	- - -			
Trauma (THS)	ns		ns			
Posttraumatic Stress (SPTSS)	ns					
Core Beliefs (CBI)			+++			
Meaning In Life (MLQ)	+++	+++				
Positive Reframing (Brief COPE subscale)						++
Urge to Talk (DTQ)				+		
Reluctance to Talk (DTQ)				-		
Actual Self-Disclosure				++		
Coping Self-Efficacy (CSE)	+++	+++				
Spiritual Coping (Brief COPE subscale)						++
Perceived Social Support (MSPSS)						++
Hospitalised for psychosis						+
Resilience (CYRM or ARM)						++
Recovery (RAS)						++

ARM: Adult Resilience Measure - Brief; CBI: Core Beliefs Inventory; CSE: Coping Self-Efficacy; CYRM: Child and Youth Resilience Measure - Brief; DTQ: Disclosure of Trauma Questionnaire; FEP: First-Episode Psychosis; MLQ: Meaning in Life Questionnaire; MSPSS: Multidimensional Scale of Perceived Social Support; PANSS: Positive, Negative and Stress Symptoms; QPR: Process of Recovery Questionnaire; RAS: Recovery Assessment Scale; SPTSS: Screen for Posttraumatic Stress Symptoms; THS: Trauma History Screen

^a^Paper number as presented in Online Supplement 2

Positive associations: + = p<0.05, ++ = p<0.01, +++ = p<0.001

Negative associations: - = p<0.05, - - = p<0.01, - - - = p<0.001

ns = tested but not significant

Cross-sectionally, PTG was positively associated with meaning in life, positive reframing, urge to talk, actual self-disclosure, coping self-efficacy, spiritual coping, perceived social support, hospitalisation for psychosis, resilience, core beliefs, and recovery, and negatively associated with a total score on the Positive, Negative Stress Symptoms Scale (PANSS) and its subscales. Coping self-efficacy is defined as an individual’s confidence in their ability to cope effectively [24]. Covariates tested but not significantly correlated with total PTGI scores included measures of psychosis using the Scale for the Assessment of Positive Symptoms (SAPS), Scale for the Assessment of Negative Symptoms (SANS), post-traumatic stress, traumatic history, time since diagnosis, negative impact of FEP, age, years of education, gender and family status.

## Objective 2: Associations independent of known covariates

Two studies (papers #14 and #37) conducted regression analyses examining 12 variables and are presented as Table 2.

**Table 2:** Regression analyses (n=2)

	Sample 2	Sample 3
**Paper Number^**	**14**	**37**
**Sample Size**	34	94
**PTG Scale used**	PTGI	PTGI
**Objective 1: Association**		
PTSD Symptoms	+++	
Recovery (QPR)	+++	
Actual Self-Disclosure	+	
Reluctance to Talk	-	
Urge to Talk	ns	
Hospitalised for psychosis		+++
Negative impact of FEP		ns
Resilience		ns
Social Support		ns
Spiritual Coping		+++
Positive Reframing		++
Recovery (RAS)		++

^Paper number as presented in Online Supplement 2

Positive associations: + = p<0.05, ++ = p<0.01, +++ = p<0.001

Negative associations: - = p<0.05

ns = tested but not significant

Mediation analyses were conducted by four studies (papers #9, #10, #11 and #14) and are presented as Table 3.

**Table 3:** Mediation analyses with total PTGI score as an outcome (n=4)

Outcome: Total PTGI Score	Mediators			
	Sample 1			Sample 2
Exposure	Meaning in Life [9, 10]	Coping Self-Efficacy [9, 10]	Core Beliefs Inventory [11]	Recovery [14]
**PANSS Total Score**	++	+	++	
**PANSS Positive Symptoms**	-	-	++	
**PANSS Negative Symptoms**	++	+	++	
**PANSS General Symptoms**	++	+	++	
**Traumatic History**	-	-	++	
**Posttraumatic Stress**	++	+		
**Actual Self-Disclosure**				+*

NB: ++ = Full Mediation; + = Partial Mediation; +*= Significant Mediator; - = Non-Significant Mediator

One example of a significant mediator is provided by meaning in life, which mediated the relationship between psychosis symptomatology and PTG. PTG was also associated with psychosis symptomatology, post-traumatic stress, and actual self-disclosure, when considering an individual’s level of meaning in life, coping self-efficacy, core beliefs, and recovery. PTG was only associated with PANSS positive symptoms and traumatic history when considering core beliefs.

### Objective 3: Temporal relationship between PTG and psychosis

No studies examining the temporal relationship over time between PTG and psychosis were included.

### Objective 4: Facilitators of PTG

#### Stage 1 (Developing a preliminary synthesis)

A total of 33 studies involving 404 unique participants were included in the narrative synthesis (papers #15 and #16 have the same sample). The thematic analysis identified seven superordinate themes illustrating the facilitators of PTG. These include (1) Personal identity and strength, (2) Receiving support, (3) Opportunities and possibilities, (4) Strategies for coping, (5) Perspective shift, (6) Emotional experience, and (7) Relationships, organised to give the acronym PROSPER, as shown in Table 4.

**Table 4:** Facilitators of posttraumatic growth in people with psychosis (33 studies, 230 participants)

		Total	Recruitment			Participants	
	Theme and Sub-theme	Proportion n (%)	Mental Health Services n (%)	Other n(%)	Mixed n (%)	First Episode n (%)	Non-First Episode n (%)
	Number of studies	33	27	9	1	5	28
**P**	**1. Personal Identity and Strength** 1.1 Self-Efficacy 1.2 Identity Development	**26 (78.8)**	**22 (66.7)**	**5 (15.2)**	**0 (0)**	**5 (15.2)**	**22 (66.7)**
		18 (54.5)	15 (45.5)	3 (9.1)	0 (0)	4 (12.1)	13 (39.4)
		22 (66.7)	17 (77.3)	5 (15.2)	0 (0)	4 (12.1)	17 (51.5)
**R**	**2. Receiving Support** 2.1 Therapeutic approaches 2.2 Support	**20 (60.6)**	**14 (42.4)**	**6 (18.2)**	**0 (0)**	**4 (12.1)**	**15 (45.5)**
		10 (30.3)	6 (18.2)	4 (12.1)	0 (0)	0 (0)	10 (30.3)
		16 (48.5)	12 (36.4)	4 (12.1)	0 (0)	4 (12.1)	11 (33.3)
**O**	**3. Opportunities and Possibilities**	**22 (66.7)**	**19 (57.6)**	**3 (9.1)**	**0 (0)**	**4 (12.1)**	**18 (54.5)**
**S**	**4. Strategies for Coping** 4.1 Coping Strategies 4.2 Skill Development 4.3 Disclosure	**23 (69.7)**	**15 (45.5)**	**7 (21.2)**	**1 (3.0)**	**5 (15.2)**	**17 (51.5)**
		15 (45.5)	9 (27.3)	5 (15.2)	1 (3.0)	3 (9.1)	12 (36.4)
		7 (21.2)	6 (18.2)	1 (3.0)	0 (0)	2 (6.1)	5 (15.2)
		7 (21.2)	3 (9.1)	4 (12.1)	0 (0)	2 (6.1)	4 (12.1)
**P**	**5. Perspective Shift**	**22 (66.7)**	**17 (51.5)**	**5 (15.2)**	**0 (0)**	**4 (12.1)**	**18 (54.5)**
**E**	**6. Emotional Experience** 6.1 Enhancing Emotional Experience 6.2 Seeking Information 6.3 Empathy and Compassion	**14 (42.4)**	**11 (33.3)**	**2 (6.1)**	**1 (3.0)**	**4 (12.1)**	**10 (30.3)**
		10 (30.3)	8 (24.2)	1 (3.0)	1 (3.0)	2 (6.1)	8 (24.2)
		3 (9.1)	3 (9.1)	0 (0)	0 (0)	1 (3.0)	2 (6.1)
		5 (15.2)	4 (12.1)	1 (3.0)	0 (0)	1 (3.0)	4 (12.1)
**R**	**7. Relationships**	**15 (45.5)**	**9 (27.3)**	**5 (15.2)**	**1 (3.0)**	**3 (9.1)**	**11 (33.3)**

Definitions and examples of the PROSPER superordinate themes and sub-themes are presented in Online Supplement 3. Vote counting indicated that more than half of the papers discussed five of the seven facilitators including *Personal identity and strength, Receiving support, Opportunities and possibilities, Strategies for coping*, and *Perspective shift* as mechanisms supporting PTG.

#### Stage 2 (Comparison between studies)

Studies recruiting from mental health services (n=27), other settings (n=9), and mixed settings (n=1) were compared. Including only the mental health service samples did not lead to the removal of any PROSPER dimension, or significant changes to the frequency of themes. Inclusion of the nine non-service samples did not lead to the exclusion of any theme, but the frequency in which themes appeared changed, with strategies for coping and receiving support identified as the most endorsed themes. Studies involving people with first-episode psychosis (n=5) versus multiple episodes of psychosis (n=28) were compared, again the content of the PROSPER framework did not change in either group, however people with multiple episodes or long-term psychosis commonly endorsed personal identity and strength, opportunities and possibilities and perspective shift as facilitators of PTG.

#### Stage 3 (Sensitivity analysis)

The quality rating, shown in Online Supplement 4, identified 35 studies as high quality and 2 as low quality. Including just the high-quality studies did not change the framework content or the frequency of theme ordering.

## Discussion

This systematic review and narrative synthesis identified significant correlates and predictors of PTG and developed the PROSPER framework characterising the facilitators of PTG in psychosis. The facilitators in the PROSPER framework were identified across all included studies.

The facilitators of PTG in people with psychosis were consistent with other research examining PTG in psychosis and other severe mental health problems [7, 14]. The using *personal identity and strength* theme was the most frequently endorsed theme promoting PTG in psychosis. Identity development is a key process implicated in personal recovery [25]. Five ways of conceptualising identity changes in psychosis have been identified from a systematic review:1) characteristics of psychosis, 2) altered cognitive functioning, 3) internalised stigma, 4) lost roles and relationships, and 5) personal growth [26]. Such influences highlight the different ways people with psychosis may understand and make meaning from their experiences. Differing conceptualisations of psychosis have been proposed by organisations such as the Hearing Voices Movement and in academic disciplines such as Mad Studies. Development of subjective explanatory frameworks for one’s experience which are personally helpful can support meaning making processes, allowing for adaptation and reconstruction of personal narratives.

*Emotional experience* was the least frequently reported facilitator of PTG from the included studies. This contrasts with the emphasis on symptom reduction in existing interventions and mental health services. Whilst this may be attributed to the focus of the included studies, namely PTG [8] and personal recovery [27], it nevertheless indicates that PTG in psychosis extends beyond the distress of symptoms experienced. Meaning in life, coping self-efficacy and core beliefs were all identified as significant mediators of the relationship between total PANSS scores and PTG. This is in line with PTG theory which suggests that PTG extends beyond the distress that is associated with the adversity such that it is the experience of adversity which creates the ‘disruption of one’s assumptive world’, leading to the post-traumatic re-evaluation of one’s core beliefs as the aftermath [8].

A higher level of self-perceived recovery (i.e. personal recovery) was associated with PTG and identified as a significant mediator between actual self-disclosure and PTG. This may indicate that people with psychosis who are earlier in their recovery journey may find it more difficult to experience PTG compared with individuals at a later stage of recovery. Further studies are required to delineate whether recovery mediates the relationship between symptoms of psychosis and PTG, to ascertain if being at a certain stage of recovery is necessary for PTG.

Mediators of PTG identified in the review require higher order cognitive processing skills, such as metacognitive processing. Given that cognitive difficulties are a core feature of psychosis [28, 29], it is plausible that people who are at further along in their recovery journey may be better equipped to engage in higher order cognitive processes. Future research could examine investigate whether a certain level of cognitive functioning is required to experience PTG.

Interventions focused on broadly improving coping strategies and promoting meaning-making have both been proposed within the literature [30]. This could be integrated with the promotion of *personal identity and strengths* to target additional aspects associated with PTG. Whilst interventions to support PTG have been evaluated in other populations such as cancer [31, 32], however, no intervention has been developed to promote PTG in people with psychosis. Greater clarification of when it is most effective to introduce such an intervention is also required, both in relation to clinical presentation and stage of recovery. Yet, the identification of the emotional experience may indicate that people continue to struggle with trauma and supports suggestions that ‘trauma-related’ growth may better encapsulate the experiences of people living with psychosis [7].

In this review PTG is conceptualised as an individualistic phenomenon and most of the findings reflect intrapersonal changes. The review’s findings highlight the importance of interpersonal and social factors (such as social support and mental health services) to the recovery of individuals with psychosis. Other factors such as public and internalised stigma have been implicated in affecting the recovery of people with psychosis [33, 34]. There is a need for future research to clarify the role and impact of social and environmental factors in the promotion of PTG, to better support the re-orientation of mental health and social care services.

### Strengths and limitations

The strength of this paper is in its use of a systematic review design and the screening of papers from multiple languages representing a broad geographic region. In addition, the use of several analysts with differing expertise, including lived experience of PTG, ensured a diversity of perspectives that was drawn upon throughout the review. This review however is not without limitations. First, despite the screening of papers in seven languages, only papers published in English met the review inclusion criteria. The predictors of PTG in non-psychiatric populations differed depending on country, indicating the potential impact of cultural factors influencing the experience of PTG [29]. Whilst this is representative of the bias towards the publication of English language papers in the wider research literature, the generalisability of the review’s findings may only be applicable to cultures in the global North [35]. Second, only six cross-sectional quantitative papers, reporting three cohorts, were included in the review. Whilst this may reflect the review’s novel application of PTG to individuals with psychosis, it signals that further primary research is required to replicate and confirm the findings. All quantitative studies used the same outcome measure, but different measures were used to explore predictor variables. Consistency in the use of patient-reported outcome measures may allow for better comparison of cohorts, leading to a more comprehensive understanding. For example, the use of the PANSS led to the identification that higher psychosis symptoms were associated with lower PTG scores [36], yet use of the SANS and SAPS did not lead to the identification of a relationship [37]. Therefore, the findings provide a preliminary understanding of how PTG in psychosis occurs. However, it should be noted the differences in sampling, such that there may be important differences between the experience of PTG in people with first-episode and multiple or long-term psychosis. Additionally, all quantitative studies were cross-sectional in nature and did not provide evidence for the temporal relationship. Multivariate repeated measures designs are required to delineate the temporal relationship between PTG and psychosis. This is an important knowledge gap since a model of causality can inform staging of clinical interventions. Third, it is known that not everyone will experience PTG [8], however what is unclear is what proportion of individuals with psychosis experience PTG. Future cohort studies are required to identify prevalence rates and individual and environmental predictors of PTG. Fourth, whilst the MMAT led to the identification of two papers that were of low quality, it did not allow for the exclusion of any papers. This may be attributed to the broad categories used to screen papers. Future research might use screening measures that are specific to the methodologies used in included papers. Fifth, only three psychiatric journals were used in the hand searching of journals. Despite the robust search of ten databases, papers published within trauma-focused journals may have been missed.

## Conclusion and implications

Post-traumatic growth can occur in individuals with experience of psychosis, manifesting across seven domains. Given the high rates of trauma, PTSD comorbidity, and frequently reported negative experiences when seeking and during treatment among individuals with psychosis, a clinical focus on facilitating growth from negative and traumatic experiences is indicated. Despite increasing psychological interventions for people with psychosis, these have primarily focused on the treatment of symptoms [38]. There is a lack of research surrounding interventions promoting positive changes in people with psychosis. Both identity and cognitive processes have been implicated in the experience of PTG. Interventions targeting both identity and cognitive processes may support PTG in people with psychosis. An individual’s identity is embedded within the personal narratives told to oneself and others. These personal narratives are constructed by connecting past, present and future events, and the associated cognitive, affective, and self-representations [39]. However, people with psychosis can find the construction and narration of a cohesive personal narrative challenging [40]. Narrative therapies focus on the construction of personal stories to support the narration of one’s evolving life story. Cognitive-behavioural interventions can enhance of coping strategies and cognitive processing requirements. The integration of existing narrative and cognitive-behavioural therapies for use in people with psychosis may be helpful to support identity development. One example is Narrative Enhancement Cognitive Therapy which is a group-based manualised intervention focused on reducing self-stigma in people with severe mental health problems [41]. Adaptation and delivery of such an intervention through online platforms could include PTG oriented self-management exercises and allows for engagement from a larger number and reach of individuals.

The identification of the importance of identity processes in promoting PTG in people with psychosis also supports the case for a reorientation of mental health services from a symptomatology focus towards assisting individuals to develop a positive identity. However, this raises the question of who is responsible for fostering a positive identity in people with psychosis? This may involve more focus on avoiding the imposition of a clinical explanatory model to support personal meaning-making [26]. Systemically, the integration of supportive models that exist outside of formal mental health services (e.g., Hearing Voices Movement network) can complement the work of mental health services and provide individuals with a choice in conceptualising their experiences. This may cultivate a more personally meaningful identity that encourages a narration involving a stronger sense of self. Peer support workers are in a unique position to discuss and share their own experiences of growth and identity with others. Integration of knowledge about who and when to talk about growth may be useful within peer support training programs.

## Supplementary Information

**Additional file 1.**

**Additional file 2.**

**Additional file 3.**

**Additional file 4.**

## Data Availability

Data extracted as part of the systematic review are available as online supplements.

## Abbreviations

FEP: First Episode Psychosis
MMAT: Mixed Methods Appraisal Tool
PANSS: Positive, Negative Stress Symptoms Scale
PRISMA: Preferred Reporting Items for Systematic Reviews and Meta-Analyses
PROSPERO: International Prospective Register of Systematic Reviews
PTG: Post- Traumatic Growth
PTGI: Post-Traumatic Growth Inventory
SANS: Scale for the Assessment of Negative Symptoms
SAPS: Scale for the Assessment of Positive Symptoms
