# Supplementary material for: Post-traumatic growth in psychosis: a systematic review and narrative synthesis
Source: BMC Psychiatry. 2021 Dec 6;21:607. doi: 10.1186/s12888-021-03614-3 (PMC8647418; doi:10.1186/s12888-021-03614-3)
Supplement: Supplementary file 1 — Additional file 1. [file 12888_2021_3614_MOESM1_ESM.docx]

**Online Supplement 1. Search Terms**

Search strategy used for MEDLINE

1 exp Posttraumatic Growth, Psychological/ (124)

2 ("posttraumatic growth" or "post-traumatic growth" or "post traumatic growth" or "traumatic growth" or "stress-related growth" or "stress related growth" or thriving or "benefit finding" or "benefit-finding" or "finding benefit" or "perceived benefit" or "perceived-benefit" or "positive by product" or "positive-by-product" or "meaning" or "meaning-making" or "meaning making" or "positive change" or "adversarial growth" or "adversial growth" or "positive adjustment" or "positive illusion" or "positive psychological change" or "self-perceived posttraumatic growth" or "illusory growth" or "positive adaptation").tw,kw. (60031)

3 ("posttraumatic growth inventory" or "post-traumatic growth inventory" or "post traumatic growth inventory" or "PTGI" or "posttraumatic growth inventory-42" or "PTGI-42" or "posttraumatic growth inventory for children" or "PTGI-C-R" or "benefit findings scale" or "benefit-finding scale" or "BFS" or "Stress-related growth scale" or "stress related growth scale" or "SRGS" or "personal growth initiative scale" or "PGIS").tw,kw. (2059)

4 1 or 2 or 3 (61674)

5 exp Psychotic Disorders/ (51444)

6 exp Schizophrenia/ (103422)

7 exp Schizophrenic Psychology/ (33133)

8 (psychosis or psychoses or psychotic or schizophrenic or schizophrenia or madness or hallucinations or "spiritual awakening" or "spiritual emergenc*" or "spiritual crisis" or "First-episode psychosis" or "first episode psychosis" or "early onset psychosis" or "early-onset psychosisor schizoaffective disorder" or "schizophreniform disorder" or "delusional disorder" or "delusion").tw,kw. (172456)

9 5 or 6 or 7 or 8 (209656)

10 4 and 9 (1181)

11 limit 10 to english language (967)

12 limit 11 to yr="1995 -Current" (783)
