# Supplementary material for: Post-traumatic growth in psychosis: a systematic review and narrative synthesis
Source: BMC Psychiatry. 2021 Dec 6;21:607. doi: 10.1186/s12888-021-03614-3 (PMC8647418; doi:10.1186/s12888-021-03614-3)
Supplement: Supplementary file 2 — Additional file 2. [file 12888_2021_3614_MOESM2_ESM.docx]

**Online Supplement 2. Data abstraction table characterising included studies (n=37)**

| **Paper Number** | **Full Citation** | **Language** | **Country** | **Design** | **Participants** | **Sample Size** | **Setting** | **FEP or Non-FEP** | **Diagnosis** |
| --- | --- | --- | --- | --- | --- | --- | --- | --- | --- |
| 1 | Connell M, Schweitzer R, R. K. Recovery from first episode psychosis and recovering self: A qualitative study. Early Intervention in Psychiatry. 2014;38(4):359-64. | English | Australia | Qualitative | Service Users | 20 | Community MH service | FEP | Other – Psychotic Disorder (100%) |
| 2 | Dos Santos B, V. B. Qualitatively exploring hearing voices network support groups. Journal of Mental Health Training Education and Practice. 2015;10(1):26-38. | English | Australia | Qualitative | Service Users | 4 | Hearing Voices Group | Non-FEP | Schizophrenia (75%)  Bipolar (25%) |
| 3 | Dunkley JE, Bates GW. Recovery and adaptation after first-episode psychosis: The relevance of posttraumatic growth. Psychosis: Psychological, Social and Integrative Approaches. 2015;7(2):130-40. | English | Australia | Qualitative | Service Users and Carers | SU (n=10)  Carers (n=8) | Outpatient service | FEP | Other (100%) |
| 4 | Ho RTH, Chan CKP, Lo PHY, Wong PH, Chan CLW, Leung PPY, et al. Understandings of spirituality and its role in illness recovery in persons with schizophrenia and mental-health professionals: A qualitative study. BMC Psychiatry. 2016;16(1):86 | English | Hong Kong | Qualitative | Service Users and Health Professionals | SU (n=18)  HP (n=19) | Outpatient service | Non-FEP | Schizophrenia (100%) |
| 5 | Jackson LJ, Hayward M, Cooke A. Developing positive relationships with voices: A preliminary Grounded Theory. International Journal of Social Psychiatry. 2010;57(5):487-95. | English | United Kingdom | Qualitative | Service Users | 12 | Community MH service  HVM group | Non-FEP | None reported |
| 6 | Jones M, Coffey M. Voice hearing: A secondary analysis of talk by people who hear voices. International Journal of Mental Health Nursing. 2012;21(1):50-9. | English | United Kingdom | Qualitative | Service Users | 20 | Community MH service | Non-FEP | Schizophrenia (75%)  Psychosis (10%)  Bipolar (5%)  Does not say (10%) |
| 7 | Jordan G, Malla A, Iyer S. "It's brought me like a lot closer to who i am": Aspects and facilitators of positive change following a first episode of psychosis. Early Intervention in Psychiatry. 2019;12 (Supplement 1):72. | English | Canada | Mixed Methods | Service Users | Quant n=94  Qual n=12 | Community MH service | FEP | Does not specify |
| 8 | Mapplebeck C, Joseph S, Sabin-Farrell R. An Interpretative Phenomenological Analysis of Posttraumatic Growth in People With Psychosis. Journal of Loss & Trauma. 2015;20(1):34-45. | English | United Kingdom | Qualitative | Service Users | 7 | Support group | Non-FEP | Schizophrenia (100%) |
| 9 | Mazor Y, Gelkopf M, Roe D. Posttraumatic growth among people with serious mental illness, psychosis and posttraumatic stress symptoms. Comprehensive Psychiatry. 2018;81:1-9. | English | Israel | Cross-sectional | Service Users | 121 | Community MH service | Non-FEP | Schizophrenia or schizoaffective (78.5%)  Bipolar with past psychotic symptoms (7.4%)  Personality or affective disorders with psychotic symptoms (14.1%) |
| 10 | Mazor Y, Gelkopf M, Mueser KT, Roe D. Posttraumatic growth in Psychosis. Frontiers in Psychiatry. 2016;7. | English | Israel | Cross-sectional | Service Users | 121 | Community MH service | Non-FEP | Schizophrenia or schizoaffective (78.5%)  Bipolar with past psychotic symptoms (7.4%)  Personality or affective disorders with psychotic symptoms (14.1%) |
| 11 | Mazor Y, Gelkopf M, Roe D. Posttraumatic Growth in Psychosis: Challenges to the Assumptive World. Psychological Trauma: Theory, Research, Practice & Policy. 2019;12(1):3-10. | English | Israel | Cross-sectional | Service Users | 121 | Community MH service | Non-FEP | Schizophrenia or schizoaffective (78.5%)  Bipolar with past psychotic symptoms (7.4%)  Personality or affective disorders with psychotic symptoms (14.1%) |
| 12 | Murphy MA. Coping with the spiritual meaning of psychosis. Psychiatric Rehabilitation Journal. 2000;24(2):179-83. | English | United States | Qualitative | Service Users | 8 | Clubhouse | Non-FEP | Schizophrenia (75%)  Schizoaffective (12.5%)  Bipolar (12.5%) |
| 13 | Ng RMK, Pearson V, Lam M, Law CW, Chiu CPY, Chen EYH. What does recovery from schizophrenia mean? Perceptions of long-term patients. International Journal of Social Psychiatry. 2008;54(2):118-30. | English | Hong Kong | Qualitative | Service Users | 8 | Clubhouse | Non-FEP | Schizoaffective disorder  Schizophrenia |
| 14 | Pietruch M, Jobson L. Posttraumatic growth and recovery in people with first episode psychosis: An investigation into the role of self-disclosure. Psychosis: Psychological, Social and Integrative Approaches. 2012;4(3):213-23. | English | United Kingdom | Cross-sectional | Service Users | 34 | Community MH service | FEP | Does not specify |
| 15 | Windell D, Norman R, Malla AK. The personal meaning of recovery among individuals treated for a first episode of psychosis. Psychiatric Services. 2012;63(6):548-53. | English | Canada | Qualitative | Service Users | 30 | Community MH service | FEP | Schizophrenia (53%)  Schizoaffective (27%)  Psychosis not otherwise specified (10%)  Substance induced psychosis (7%)  Bipolar I with psychotic features (3%) |
| 16 | Windell DL, Norman R, Lal S, Malla A. Subjective experiences of illness recovery in individuals treated for first-episode psychosis. Social Psychiatry & Psychiatric Epidemiology. 2015;50(7):1069-77. | English | Canada | Qualitative | Service Users | 30 | Community MH service | FEP | Schizophrenia (53%)  Schizoaffective (27%)  Psychosis not otherwise specified (10%)  Substance induced psychosis (7%)  Bipolar I with psychotic features (3%) |
| 17 | Connell M, Schweitzer R, King R. Recovery from first-episode psychosis: A dialogical perspective. Bulletin of the Menninger Clinic. 2015;79(1):70-90. | English | Australia | Qualitative | Service Users | 12 | Specialist Early Psychosis Service | FEP | Bipolar 1 (41.7%)  Schizophrenia or (16.7%)  Schizophrenia (25%)  Drug induced psychosis (16.7%) |
| 18 | de Wet A, Swartz L, Chiliza B. Hearing their voices: The lived experience of recovery from first-episode psychosis in schizophrenia in South Africa. International Journal of Social Psychiatry. 2015;61(1):27-32. | English | South Africa | Qualitative | Service Users | 7 | Community MH Service | Non-FEP | Paranoid Schizophrenia (42.6%)  Schizophreniform (28.6%)  Schizophrenia (28.6%) |
| 19 | Eisenstadt P, Monteiro VB, Diniz MJA, Chaves AC. Experience of recovery from a first-episode psychosis. Early Intervention in Psychiatry. 2012;6(4):476-80. | English | Brazil | Qualitative | Service Users | 16 | Community MH Service | FEP | Paranoid Schizophrenia (50%)  Schizoaffective disorder (18.8%)  Catatonic schizophrenia (6.3%)  Schizophreniform (12.5%)  Persistent delusional disorder (8.3%) |
| 20 | Hirschfeld R, Smith J, Trower P, Griffin C. What do psychotic experiences mean for young men? A qualitative investigation. Psychology and Psychotherapy: Theory, Research and Practice. 2005;78:249-70. | English | United Kingdom | Qualitative | Service Users | 6 | Community MH service | Non-FEP | Schizophrenia (100%) |
| 21 | Subandi MA. Bangkit: The processes of recovery from first episode psychosis in Java. Culture, Medicine, and Psychiatry. 2015;39(4):597-613. | English | Indonesia | Qualitative | Service Users | 7 | Hospital and Community Setting | FEP | Does not specify |
| 22 | Clements S, Coniglio F, L. M. "I'm not telling an illness story. I'm telling a story of opportunity": Making sense of voice hearing experiences. Community Mental Health Journal 2020;56(2):196-205. | English | Australia | Qualitative | Service Users | 5 | Hearing Voices Support Group | Non-FEP | Schizophrenia (80%)  Schizoaffective disorder (20%) |
| 23 | Dixon L, Sanderson C, Alexander T, L. H. A weird but interesting journey: Personal traumatic growth for individuals with hallucinations. Jounral of Psychology and Psychotherapy. 2018;8(3):1-6 | English | United Kingdom | Qualitative | Service Users | 7 | Community MH Services | Non-FEP | Other – Hallucinations (100%) |
| 24 | Drinnan A, T. L. Deconstructing delusions: A qualitative study examining the relationship between religious beliefs and religious delusions. Mental Health, Religion & Culture. 2006;9(4):317-31. | English | United Kingdom | Qualitative | Service Users | 7 | Community MH Services | Non-FEP | Does not specify |
| 25 | Hanevik H, Hestad KA, Lien L, Joa I, Larsen TK, LJ. D. Religiousness in first-episode psychosis. Archiv fur Religions psychologie / Archive for the Psychology of Religion. 2017;39(2):139-64. | English | Norway | Qualitative | Service Users | 18 | Community MH Services | FEP | Does not specify (100%) |
| 26 | Heffernan S, Neil S, Thomas Y, S. W. Religion in the recovery journey of individuals with experience of psychosis. Psychosis: Psychological, Social and Integrative Approaches. 2016;8(4):346-56. | English | United Kingdom | Qualitative | Service Users | 10 | Community MH Services | FEP | Does not specify (100%) |
| 27 | Nixon G HB, Peters T. . Recovery from psychosis: A phenomenological inquiry. International Journal of Mental Health and Addiction. 2010;8:620-35. | English | Canada | Qualitative | Service Users | 17 | Community MH Service | Non-FEP | Does not specify |
| 28 | Smith S, MJ. S. Religious and/or spiritual practices: extending spiritual freedom to people with schizophrenia. Canadian Journal of Occupational Therapy - Revue Canadienne d Ergotherapie. 2012;79(2):77-85. | English | Canada | Qualitative | Service Users | 9 | Community MH Service | Non-FEP | Schizophrenia (100%) |
| 29 | Ng P, Chun RWK, A. T. Recovering from hallucinations: A qualitative study of coping with voices hearing of people with schizophrenia in Hong Kong. The Scientific World Journal. 2012:1-8. | English | Hong Kong | Qualitative | Service Users | 20 | Community NGO Service | Non-FEP | Schizophrenia (100%) |
| 30 | Nowak I, Waszkiewicz J, Switaj P, Sokol-Szawlowska M, M. A. A Qualitative Study of the Subjective Appraisal of Recovery Among People with Lived Experience of Schizophrenia in Poland. Psychiatric Quarterly. 2017;88:435-46. | English | Poland | Qualitative | Service Users | 28 | Inpatient ward and Community MH Service | Non-FEP | Schizophrenia (100%) |
| 31 | Beavan V, de Jager A, B. dS. Do peer-support groups for voice-hearers work? A small scale study of Hearing Voices Network support groups in Australia. Psychosis: Psychological, Social and Integrative Approaches 2017;9(1):57-66. | English | Australia | Mixed-method (NB: only qualitative findings included in the review) | Service Users | 29 | Hearing Voices Group | Non-FEP | Does not specify |
| 32 | Oakland L, K. B. "Lifting the veil": A qualitative analysis of experiences in Hearing Voices Network groups. Psychosis: Psychological, Social and Integrative Approaches. 2015;7(2):119-29. | English | United Kingdom | Qualitative | Service Users | 11 | Support groups | Non-FEP | Does not specify |
| 33 | Sinha N, S. R. Living with voices: A thematic analysis of individuals' experiences of voice-hearing in india. . Psychosis: Psychological, Social and Integrative Approaches. 2020;12(2):115-27. | English | India | Qualitative | Service Users | 27 | Inpatient Ward and Community MH Service | Non-FEP | Schizophrenia (40.7%)  Depression with psychotic features (25.9%)  PTSD (22.2%)  Drug induced psychosis (11.1%) |
| 34 | Roe D, Chopra M. Beyond coping with mental illness: Toward personal growth. American Journal of Orthopsychiatry. 2003;73(334-344). | English | Israel | Qualitative | Service Users | 41 | Inpatient Ward | Non-FEP | Schizophrenia (51.2%)  Schizoaffective disorder (30.2%)  Major affective disorder with psychotic features (18.6%) |
| 35 | de Jager A, Rhodes P, Beavan V, Holmes D, McCabe K, Thomas N, et al. Investigating the lived experience of recovery in people who hear voices. Qualitative Health Research. 2016;26(1409-1423). | English | Australia | Qualitative | Service Users | 11 | Hearing Voices Group | Non-FEP | Schizophrenia (36.4%)  Schizoaffective disorder (36.4%)  Psychosis not otherwise specified (27.3%) |
| 36 | Aloneftis R, Challenor J. 'We're not all dangerous and crazy'. Negotiating the voice hearing identity: A critical discursive approach. Journal of Health Psychology. 2019. | English | United Kingdom | Qualitative | Service Users | 8 | Hearing Voices Group | Non-FEP | Does not specify (100%) |
| 37 | Jordan G, Malla A, Iyer SN. Perceived facilitators and predictors of positive change and posttraumatic growth following a first episode of psychosis: A mixed methods study using a convergent design. BMC Psychiatry. 2020;20(289):1-16. | English | Canada | Mixed-methods | Service Users | Quantitative (n=94)  Qualitative (n=12) | Community MH Service | FEP | Schizophrenia (64.9%)  Affective psychosis (35.1%) |
