# Supplementary material for: Post-traumatic growth in psychosis: a systematic review and narrative synthesis
Source: BMC Psychiatry. 2021 Dec 6;21:607. doi: 10.1186/s12888-021-03614-3 (PMC8647418; doi:10.1186/s12888-021-03614-3)
Supplement: Supplementary file 3 — Additional file 3. [file 12888_2021_3614_MOESM3_ESM.docx]

**Online Supplement 3. The PROSPER framework: Definitions and examples of the facilitators of PTG in psychosis**

| **Theme** | **Sub-theme** | **Definition** | **Incorporated themes identified in included papers** | **Illustrative quotes** | **Paper References** |
| --- | --- | --- | --- | --- | --- |
| 1. Personal Identity and Strength | 1.1 Self-Efficacy | Enhancing an individual’s belief in oneself. | Resilience  Agency  Control  Self-confidence  Self-mastery  Inner strength  Empowerment | *I feel relaxed, I feel at ease, I feel comfortable within myself, I don’t feel as scared [#2]*  *I guess it took a bit of confidence to think ‘well I might be comfortable with where things are now, but I’m miserable, so yeah, things have to change [#17]* | #2, #3, #7, #8, #15, #17, #18, #19, #20, #21, #23, #25, #26, #28, #30, #31, #33, #35, #37 |
|  | 1.2 Identity Development | The development of one’s sense of self and personal narrative. This can be through the integration of psychosis experience into identity, being able to differentiate between self and illness, self-acceptance, acceptance of illness or lifespan developmental experiences. | Differentiating self and illness  Acceptance of illness  Integrating psychosis Experiences into identity  Developing a personal narrative  Developing sense of self  Self-acceptance  Life experiences | *The experience has very much helped to understand myself and find my voice and to really get in touch with what was important to me in terms of my values, what I want to create in myself and my life.*[#22] | #1, #2, #3, #4, #6, #8, #15, #16, #20, #22, #23, #24, #25, #26, #27, #29, #30, #31, #35, #37 |
| 2. Receiving Support | 2.1 Therapeutic Approaches | Intervention or support approaches engaged in by the individual. This can include traditional treatments such as psychological or pharmacological treatments or spiritual practices such as shamanic healing or spiritual directors. | Talking therapies  Medications  Support groups  Non-traditional healers  Holistic activities (e.g. yoga and meditation) | *Some said (in one of the YouTube videos) that they can deal with this (voices) without treatment. So, for one year or so, I tried to handle it on my own. I didn’t tell anyone about it. But it will disturb me in my work, especially the bad voice. It will not let me do anything, and I didn’t know what to do to get rid of it. So, I came to a doctor for help.* [#33] | #12, #13, #15, #16, #19, #23, #27, #31, #32, #33 |
|  | 2.2 Support | Receiving or being open to the idea of receiving support from others and services which offers mental health support (within traditional and non-traditional services) | Mental health workers  Peers  Family and friends | *I have to say too that I’m very fortunate to be with a partner for the last ten years who has been extraordinarily supportive on a number of fronts....my partner is diabetic since he was a young child, so self-management wasn’t foreign to him. And so, you know, living with someone who has to take care of himself like that really has helped me too.* [#27] | #1, #2, #3, #4, #8, #13, #15, #18, #19, #20, #21, #22, #27, #30, #31, #33, #37 |
| 3. Opportunities and Possibilities | 3.1 Making use of Opportunities and Possibilities | Change in life direction or approach to life as a result of experiences of psychosis, or the identification and engagement in activities that are personally meaningful | New possibilities and direction  Meaningful activities  Religious and spiritual practice  Changing lifestyle  Embracing life | *All of my positive changes came from not those things, but getting out of those things. That is something I will absolutely make clear is that none of the positive effects that I have in my life right now came from these awful, crappy, terrible diseases*. [#37] | #3, #6, #7, #12, #15, #17, #19, #20, #21, #23, #24, #25, #26, #27, #28, #30, #31, #34, #35, #37 |
| 4. Strategies for Coping | 4.1 Coping Strategies | Specific coping strategies identified by individuals which promoted recovery or growth. Examples include boundary setting with voices, normalising, reframing, and problem solving. | Boundary setting with voices  Problem solving  Personification  Normalising  Psychological distancing  Avoidance  Minimalising  Distraction  Reframing  Developing coping strategies | *It really opened up my mind to this as an experience that was normal in the world… Whereas before that I thought it was my shame. My shame, my fault, my illness. You know, it was all about me and me broken.* [#35] | #3, #5, #17, #18, #20, #22, #23, #28, #29, #30, #31, #33, #35, #36, #37 |
|  | 4.2 Skill Development | Development of specific abilities including the ability to self-manage symptoms associated with psychosis. | Self-management of symptoms  New skills  Goal setting | *I have been really concentrating on that, doing my work and my job and coming to understand what’s the best way for me to do my job without putting too much stress on myself, because one of the reasons that I got sick was because my job was stressful…* [#34] | #37,#21, #26, #33, #35, #4, #34 |
|  | 4.3 Disclosure | Expression of lived experiences of psychosis or mental health concerns. Expression can occur in different forms including written and spoken. | Disclosure of experiences | *When ill, she maintained a closed-off posture. She confined herself to her own room, talking mainly to herself… But as she started to recover, she began to share her illness experiences, first with Nia—the psychology student who rented a room in her house and who was considered a part of the family—and then with other family members. She also disclosed details of her private illness experiences to me, including the fact that she continued to have auditory hallucinations, despite her having made considerable progress in her social functioning by returning to university.* [#21] | #1, #2, #21, #22, #31, #32 |
| 5. Perspective Shift | 5.1 Perspective Shift | Changes to one’s worldviews, view of experiences, or taking a more self-reflective stance to experiences. | Making meaning  Shift in perspective  Hope for the future  Self-reflection  Perspective taking | *I let go of that because I realized I can’t control what they’re doing, what, or what they do, or what mistakes they make because I can’t fix it for them. The only thing I can fix is myself. So I let go of that, so. That in a way I’m better because I don’t stress about those things anymore.* [#37] | #3, #37, #8, #19, #20, #23, #25, #26, #28, #30, #31, #22, #1, #4, #6, #16, #27, #29, #12, #32, #34 |
| 6. Emotional Experience | 6.1 Enhancing Emotional Experience | Improving emotional experience through the reduction of symptoms and enhanced psychological wellbeing or general sense of peacefulness. This could be achieved through the ability to embrace positive emotions and understand thoughts and feelings. | Understanding of thoughts and emotions  Reduction in symptoms  Improved psychological wellbeing  Sense of Belonging  Embracing Positive emotions  Peacefulness | *I thought people were out to get me and everyone was staring at me. And I didn’t realize it was schizophrenia. It still progressed for another…two and a half years of battling—with the schizophrenia before my perception of things changed in a more healthy manner; more true to reality.*[#16] | #4, #5, #16, #19, #23, #26, #32, #34, #37 |
|  | 6.2 Seeking Information | Seeking or gaining information about psychosis. For example psychoeducation | Gaining insight  Learning more about  mental health problems | *When I would talk to [case manager] we would go over symptoms like, are you hearing voices? These make sense, because…when someone describes what you’re feeling, then you feel, yeah, that’s my problem and that’s why I picked up on the fact that I have psychosis.* [#16] | #16,#19 |
|  | 6.3 Empathy and Compassion | Being able to understand or have concern for self and others. | Greater self-compassion for others  Empathy for others | *I show more empathy maybe, more than I used to. More understanding with people with difficulties and stuff like that.* [#23] | #3, #4, #23, #30, #35 |
| 7. Relationships | 7.1 Developing and Improving Relationships | The development or improvement of relationships with others (including peers, family, friends, spiritual figures) and improving interpersonal skills. | Improving and repairing relationships with others  Developing relationships with spiritual figures  Improving relationships with voices  Openness to new relationships  Developing interpersonal skills | *When I say we have better relationships, it’s like these prior months. It’s been like two, three months I feel like we have a better relationship because we’re seeing each other way more often … She always … been good to me. And I think it’s going to be even better in the future.* [#7] | #2, #3, #5, #7, #8, #21, #22, #25, #26, #28, #29, #30, #31, #33, #35, #37 |
