# Supplementary material for: Post-traumatic growth in psychosis: a systematic review and narrative synthesis
Source: BMC Psychiatry. 2021 Dec 6;21:607. doi: 10.1186/s12888-021-03614-3 (PMC8647418; doi:10.1186/s12888-021-03614-3)
Supplement: Supplementary file 4 — Additional file 4. [file 12888_2021_3614_MOESM4_ESM.docx]

**Online Supplement 4. Quality ratings using the MMAT**

|  | **All Study Designs** | | **1. Qualitative** | | | | | **2. Quantitative Descriptive** | | | | | **3. Mixed Methods** | | | | | **Total (%)** |
| --- | --- | --- | --- | --- | --- | --- | --- | --- | --- | --- | --- | --- | --- | --- | --- | --- | --- | --- |
| **Paper ID** | **S1** | **S2** | **1.1** | **1.2** | **1.3** | **1.4** | **1.5** | **4.1** | **4.2** | **4.3** | **4.4** | **4.5** | **5.1** | **5.2** | **5.3** | **5.4** | **5.5** |  |
| 1 | Y | Y | Y | Y | Y | Y | Y | - | - | - | - | - | - | - | - | - | - | 7 (100%) |
| 2 | Y | Y | Y | Y | Y | Y | Y | - | - | - | - | - | - | - | - | - | - | 7 (100%) |
| 3 | Y | Y | Y | Y | Y | Y | Y | - | - | - | - | - | - | - | - | - | - | 7 (100%) |
| 4 | Y | Y | Y | Y | Y | Y | Y | - | - | - | - | - | - | - | - | - | - | 7 (100%) |
| 5 | Y | Y | Y | Y | Y | Y | Y | - | - | - | - | - | - | - | - | - | - | 7 (100%) |
| 6 | Y | Y | Y | Y | Y | Y | Y | - | - | - | - | - | - | - | - | - | - | 7 (100%) |
| 7 | Y | Y | - | - | - | - | - | - | - | - | - | - | Y | Y | Y | Y | Y | 7 (100%) |
| 8 | Y | Y | Y | Y | Y | Y | Y | - | - | - | - | - | - | - | - | - | - | 7 (100%) |
| 9 | Y | Y | - | - | - | - | - | Y | Y | Y | Y | Y | - | - | - | - | - | 7 (100%) |
| 10 | Y | Y | - | - | - | - | - | Y | Y | Y | Y | Y | - | - | - | - | - | 7 (100%) |
| 11 | Y | Y | - | - | - | - | - | Y | Y | Y | Y | Y | - | - | - | - | - | 7 (100%) |
| 12 | N | Y | ? | ? | ? | ? | ? | - | - | - | - | - | - | - | - | - | - | 1  (14%) |
| 13 | Y | Y | Y | Y | Y | Y | Y | - | - | - | - | - | - | - | - | - | - | 7 (100%) |
| 14 | Y | Y | - | - | - | - | - | Y | Y | Y | N | Y | - | - | - | - | - | 6  (86%) |
| 15 | Y | Y | Y | Y | N | N | N | - | - | - | - | - | - | - | - | - | - | 4  (57%) |
| 16 | Y | Y | Y | Y | Y | Y | Y | - | - | - | - | - | - | - | - | - | - | 7 (100%) |
| 17 | Y | Y | Y | Y | Y | Y | Y | - | - | - | - | - | - | - | - | - | - | 7 (100%) |
| 18 | Y | Y | Y | Y | Y | Y | Y | - | - | - | - | - | - | - | - | - | - | 7 (100%) |
| 19 | Y | Y | Y | Y | Y | N | Y | - | - | - | - | - | - | - | - | - | - | 6  (86%) |
| 20 | Y | Y | Y | Y | Y | Y | Y | - | - | - | - | - | - | - | - | - | - | 7 (100%) |
| 21 | Y | Y | Y | Y | Y | N | Y | - | - | - | - | - | - | - | - | - | - | 6  (86%) |
| 22 | Y | Y | Y | Y | Y | Y | Y | - | - | - | - | - | - | - | - | - | - | 7 (100%) |
| 23 | Y | Y | Y | Y | Y | Y | Y | - | - | - | - | - | - | - | - | - | - | 7 (100%) |
| 24 | Y | Y | Y | Y | Y | Y | Y | - | - | - | - | - | - | - | - | - | - | 7 (100%) |
| 25 | Y | Y | Y | Y | Y | N | Y | - | - | - | - | - | - | - | - | - | - | 6  (86%) |
| 26 | Y | Y | Y | Y | Y | Y | Y | - | - | - | - | - | - | - | - | - | - | 7 (100%) |
| 27 | Y | Y | Y | Y | Y | Y | Y | - | - | - | - | - | - | - | - | - | - | 7 (100%) |
| 28 | Y | Y | Y | Y | Y | Y | Y | - | - | - | - | - | - | - | - | - | - | 7 (100%) |
| 29 | Y | Y | ? | Y | Y | Y | Y | - | - | - | - | - | - | - | - | - | - | 6  (86%) |
| 30 | Y | Y | Y | Y | Y | Y | Y | - | - | - | - | - | - | - | - | - | - | 7 (100%) |
| 31 | Y | Y | - | - | - | - | - | - | - | - | - | - | N | N | Y | N | N | 3  (43%) |
| 32 | Y | Y | Y | Y | Y | Y | Y | - | - | - | - | - | - | - | - | - | - | 7 (100%) |
| 33 | Y | Y | Y | Y | Y | Y | Y | - | - | - | - | - | - | - | - | - | - | 7 (100%) |
| 34 | Y | Y | Y | Y | Y | Y | Y | - | - | - | - | - | - | - | - | - | - | 7 (100%) |
| 35 | ? | Y | Y | Y | Y | Y | Y | - | - | - | - | - | - | - | - | - | - | 6  (86%) |
| 36 | Y | Y | Y | Y | Y | Y | Y | - | - | - | - | - | - | - | - | - | - | 7 (100%) |
| 37 | Y | Y | - | - | - | - | - | - | - | - | - | - | Y | Y | Y | Y | Y | 7 (100%) |

Y=Yes; N=No; ?=Cannot be assessed; - = Not applicable.
